# TBX3 over-expression causes mammary gland hyperplasia and increases mammary stem-like cells in an inducible transgenic mouse model

**DOI:** 10.1186/1471-213X-11-65

**Published:** 2011-10-31

**Authors:** Jing Liu, Taraneh Esmailpour, Xiying Shang, Gultekin Gulsen, Andy Liu, Taosheng Huang

**Affiliations:** 1 Department of Pediatrics, Division of Human Genetics, University of California, Irvine, USA; 2 Department of Pathology, University of California, Irvine, USA; 3 Department of Radiological Sciences, University of California, Irvine, USA; 4 Department of Developmental and Cell Biology, University of California, Irvine, USA

## Abstract

**Background:**

The T-box transcription factor TBX3 is necessary for early embryonic development and for the normal development of the mammary gland. Homozygous mutations, in mice, are embryonic lethal while heterozygous mutations result in perturbed mammary gland development. In humans, mutations that result in the haploinsufficiency of TBX3 causes Ulnar Mammary Syndrome (UMS) characterized by mammary gland hypoplasia as well as other congenital defects. In addition to its role in mammary gland development, various studies have also supported a role for Tbx3 in breast cancer development. TBX3 is over-expressed in various breast cancer cell lines as well as cancer tissue and has been found to contribute to breast cancer cell migration. Previous studies have suggested that TBX3 contributes to cancer development by its ability to bypass senescence by repressing the expression of p14^ARF^-tumor suppressor. Although many studies have shown that a dysregulation of TBX3 expression may contribute to cancer progression, no direct evidence shows TBX3 causes breast cancer.

**Results:**

In this study, we created doxycycline inducible double transgenic mice (MMTV-rtTA;tet-myc-TBX3-IRES-Luciferase) to test whether TBX3 over-expression can induce tumor formation within the mammary gland. Although over-expression of TBX3, alone, did not induce tumor formation it did promote accelerated mammary gland development by increasing mammary epithelial cell proliferation. We also show that TBX3 directly binds to and represses *NFκBIB*, an inhibitor of the NF-κB pathway known to play a role in regulating cell proliferation. Lastly, we also show that the over-expression of TBX3 is associated with an increase in mammary stem-like cells.

**Conclusions:**

Overall, our data suggests that over-expression of TBX3 may contribute to breast cancer development by promoting accelerated mammary gland development through the inhibition of the NF-κB pathway and stimulation of both mammary epithelial cell and stem-like cell proliferation.

## Background

TBX3 is a member of the T-box family of genes. T-box genes are expressed during embryonic development and have been found to regulate cell specification and organogenesis [1,2]. They are also well-known for the roles they play in many human developmental syndromes [3-6]. Tbx3 is known to function as a transcriptional repressor and is required for embryonic development and for the normal development of the mammary gland [7-11]. In mice models, homozygous mutations in which the function of Tbx3 is completely lost are embryonic lethal while haploinsufficiency of Tbx3 results in significantly reduced branching of ductal trees in adult animals [12]. In humans, mutations that result in the haploinsufficiency and loss of function of TBX3 ultimately cause Ulnar Mammary Syndrome (UMS) [3,13,14]. UMS is an autosomal dominant disorder characterized by mammary gland hypoplasia and affects limb, apocrine-gland, teeth, hair, and genital development. Besides Tbx3's role in early mammary gland development, various studies have also supported a role for Tbx3 in breast cancer development. The TBX3 gene is located at the 12q24 region which is frequently amplified in a variety of malignancies including breast cancer [7,15]. Moreover, TBX3 is over-expressed in various breast cancer cell lines as well as primary breast cancer tissues [16,17]. TBX3 is mislocalized to the cytoplasm in primary breast cancer tissues and serum TBX3 protein levels were also found to be abnormally high in early stage breast cancer patients [17,18]. More recently, it has been shown that PMA-induced up-regulation of TBX3 contributes to breast cancer cell migration [19].

TBX3 has been shown to repress the expression of the tumor suppression gene p14^ARF ^[8,9,11,20] and the murine homologue p19^ARF ^[8]. The p14/19(ARF)-Mdm2-p53 pathway plays an important role in regulating cell senescence and protects cells against oncogenic transformation which leads to tumor formation [8,9,11,20]. TBX3 over-expression has been shown to immortalize mouse embryonic fibroblast cells by suppressing p19^ARF ^[8,16,21]. We have previously shown that over-expression of TBX3 represses human p14^ARF ^by recruiting HDAC 1, 2, 3 and 5 in the MCF7 breast cancer cell line [17]. In order to identify other targets of TBX3, we used chromatin immunoprecipitation-guided ligation and selection (ChIP-GLAS) promoter array. Our results showed that 430 gene promoters are bound by TBX3 in the MCF7 breast cancer cell line (unpublished data). One of the identified genes, *NFκBIB*, is an inhibitor of NF-κB. Studies have shown that NF-κB associated pathways play an important role in cell proliferation, differentiation and apoptosis [22]. Specifically, NFκBIB inhibits NF-κB by sequestering it in the cytoplasm. Activation of NF-κB occurs upon ubiquitin mediated degradation of NFκBIB proteins via serine phosphorylation by IκB kinase (IKK). Studies have shown that inhibition of NF-κB activation in mouse mammary glands lead to defective proliferation in lobuloalveolar structures during pregnancy [23], whereas elevated NF-κB activity causes mammary hyperplasia *in vivo *[24]. Furthermore, aberrant activation of NF-κB is related to breast cancer progression, including tumor initiation, proliferation, chemoresistance and tumor metastasis [25]. Taken together, these studies suggest that a dysregulation of TBX3 expression may contribute to breast cancer development.

Further supporting the notion that Tbx3 plays a role in cancer development, recent studies have shown that increased levels of TBX3 enhanced melanoma invasiveness by repressing E-cadherin expression [26]. Recent studies have shown that TBX3, a downstream target of Wnt/β-catenin in liver cancer, has also been found to be over-expressed in human hepatocellular carcinoma and heptoblastoma [27]. Knockdown of Tbx3 in rat bladder carcinoma cell lines resulted in a lower growth rate and more apoptotic cells than controls, suggesting that Tbx3 promotes cell proliferation and is a negative regulator of apoptosis [28]. Although many studies have shown that a dysregulation of TBX3 expression may contribute to cancer progression, no direct evidence shows that TBX3 causes breast cancer.

Identifying whether TBX3 directly promotes breast cancer development and the mechanism by which it does this is important for understanding mammary development as well as the perturbations that may lead to breast cancer. In the present study, we have demonstrated that over-expression of TBX3 in our doxycycline inducible mouse model promotes accelerated mammary gland development and hyperplasia by promoting mammary epithelium cell proliferation. Moreover, we have shown that NFκBIB was dramatically down-regulated in the mammary glands of doxycycline induced double transgenic mice. Although over-expression of TBX3, alone, did not cause tumor formation within the mammary gland, our data suggests that the over-expression of TBX3 may contribute to breast cancer formation through the inhibition of the NF-κB pathway and stimulation of both mammary epithelial cell and stem-like cell proliferation.

## Results

### TBX3 over-expression is induced in MMTV-rtTA; tet-myc-TBX3 mammary glands by doxycycline administration

**Figure 1:**
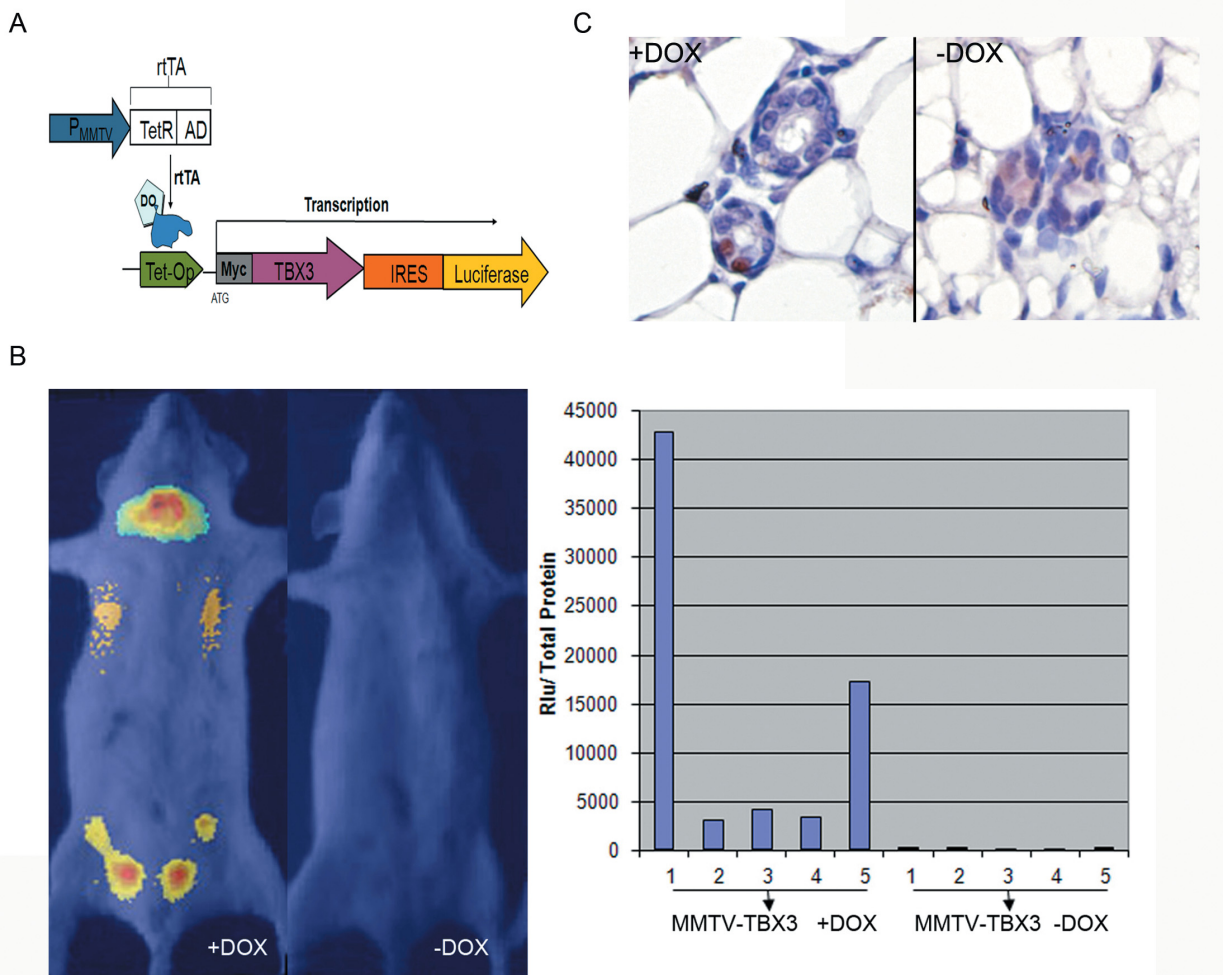
**TBX3 transgene expression is tightly controlled by doxycycline administration within mammary glands of double-transgenic mice**. A) Diagram of tetracycline inducible TBX3 transgene cassette. In the presence of doxycycline, the tetracycline reverse transcriptional activator (rtTA), expressed in mammary epithelium, binds to the tetracycline-operator (Tet-On), which in turn activates the transcription of TBX3 and firefly luciferase. B) Left panel: *In vivo *detection of luciferase reporter activity. Fifteen-week old doxycycline induced and un-induced double transgenic female mice were injected with an aqueous solution of luciferin into the peritoneal cavity. Images were taken with an ICCD camera five minutes later. Right panel: Luciferase reporter activity in tet-TBX3; MMTV-rtTA transgenic mammary glands. Mammary glands from doxycycline induced and un-induced double transgenic mice were dissected and lysates were prepared. The luciferase activity of 10μl of total protein lysate was measured. Numbers on X-axis indicate the pair of mammary gland used in the assay (n = 1). C) Immunohistochemical analysis of TBX3 expression in doxycycline induced and un-induced double transgenic littermates at 10 weeks of age. Images were captured at 40× magnification.

To construct a doxycycline inducible myc-TBX3 transgene cassette (TMILA-myc-TBX3-IRES-Luciferase), myc-TBX3 cDNA was subcloned downstream of tet operator elements (TetO) (Figure 1A). In our transgene expression cassette, the expression of the luciferase reporter gene is regulated by the same promoter as our myc-TBX3 transgene. Thus, upon induction with doxycycline, translation of the luciferase reporter gene by its own internal ribosome entry site (IRES) can be used as a marker for myc-TBX3 overexpression (Figure 1A). In order to express myc-TBX3 specifically in the mammary glands of mice, tet-myc-TBX3 mice were mated with MMTV-rtTA mice. Transgene expression was induced in double transgenic mice by adding 2mg/ml doxycycline to the drinking water. To verify that the induction of TBX3 expression within the mammary glands of mice occurred only upon the addition of doxycycline, luciferase activity was monitored by imaging the mammary glands of both doxycycline induced and un-induced double transgenic mice *in vivo*, using an ICCD camera. Prior to *in vivo *imaging, mice were sedated by intraperitoneal injection of Xylazine and Ketamine. After 5 minutes, an aqueous solution of luciferin was injected into the peritoneal cavity to detect luciferase activity and TBX3 transgene over-expression. The *in vivo *image of the doxycycline induced double transgenic mouse detected a bioluminescent signal 4-200 folds above background within all 5 pairs of mammary glands. The bright bioluminescent signal in the cervical midline of the doxycycline induced double transgenic mouse represents the first pair of mammary glands as well as leaky expression of the MMTV promoter within the salivary gland, which is frequently seen in other MMTV models (Figure 1B). No signal was detected in the age-matched un-induced double transgenic littermate control (Figure 1B, left panel). To more directly measure the luciferase activity within each mammary gland a luciferase assay was performed using tissue lysates from each mammary gland (1-5) of a single doxycycline induced double transgenic mouse (n = 1). Consistent with the *in vivo *imaging, all five mammary glands from the doxycycline induced double transgenic mice had high luciferase readings while the un-induced double transgenic littermates showed only baseline readings (Figure 1B, right panel). Direct TBX3 over-expression within the mammary gland was also detected by immunohistochemistry with an anti-TBX3 antibody. TBX3 over-expression was detected only in the induced double transgenic mouse mammary gland (Figure 1C). Endogenous TBX3 expression was not detected (Figure 1C, right panel). Overall, these results show that TBX3 over-expression is specifically induced within all 5 mammary glands of our double transgenic mice upon administration of doxycycline.

### Over-expression of TBX3 promotes accelerated mammary gland development by increasing cell proliferation

**Figure 2:**
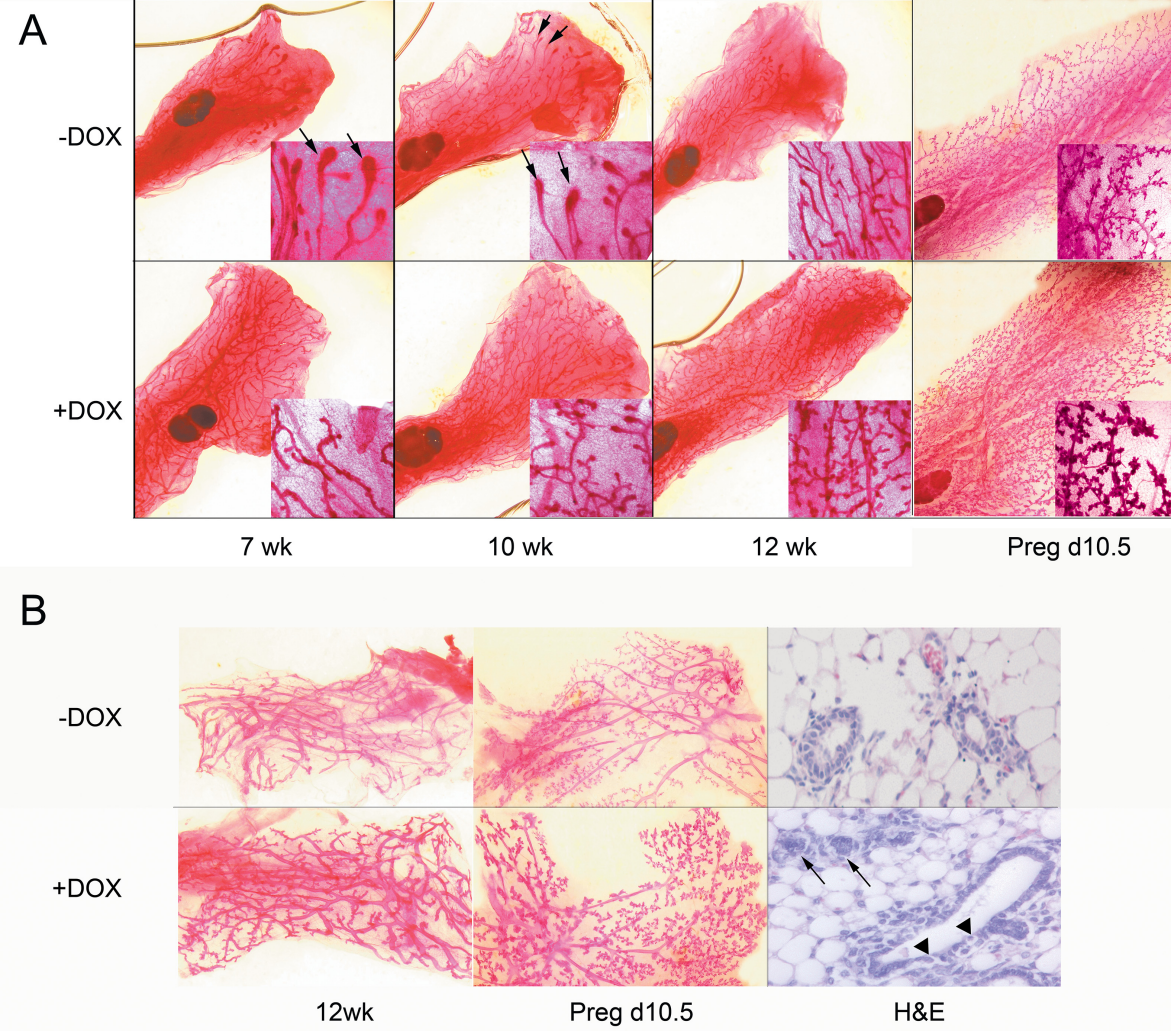
**TBX3 over-expression causes accelerated mammary gland development and hyperplasia**. A) Whole mount analysis of the 4th mammary gland from 7-weeks, 10-weeks, 12-weeks old virgin mice and day 10.5 dpc pregnant mice. Acceleration of mammary gland development is observed in doxycycline induced double transgenic mice from 7 to 12 weeks of age. TEBs are present in un-induced control mice at 7- and 10- weeks of age (arrows), whereas TEBs in doxycycline induced double transgenic mice pass the fat pad and start to disappear. At all time points, branching morphogenesis was promoted in the doxycycline induced double transgenic mice. Representative whole mount of the 4th mammary gland from 10.5 dpc pregnant un-induced double transgenic mouse has less alveoli tissue than the 4th mammary gland from day 10.5 dpc pregnant doxycycline induced double transgenic mouse (n = 3). All images were captured at 5× magnification. Image insets were captured at 20× magnification. B) Whole mount analysis of the 1st mammary gland from un-induced double transgenic mice and doxycycline induced double transgenic mice at 12 weeks of age and pregnant 10.5 dpc. Mammary glands from doxycycline induced double transgenic mice displayed enhanced side branching and advanced development of alveolar structure. Images were captured at 10× magnification. Hematoxylin and eosin stain performed on the 3rd mammary gland of doxycycline induced and un-induced double transgenic mice at 15 weeks of age showed mild hyperplasia (arrows) and disturbed organization in the mammary epithelium (arrowheads) (n = 3). Images were captured at 40× magnification.

In mice, the mammary gland development begins shortly after mid-gestation. Five pairs of mammary placodes form at the site of the future nipples [29]. These placodes invaginate and form buds within the mammary fat pad that contain few branches [29]. By birth a simple mammary ductal tree is formed that occupies a small portion of the fat pad [30]. After birth, growth of the mammary gland is relatively quiescent until puberty [31]. At puberty, club-shaped structures called the terminal end buds (TEBs) form at the tips of the ductal tree. During this period, cell proliferation in TEBs results in ductal elongation through the mammary fat pad. TEBs not only elongate through the fat pad, but also bifurcate to form new primary ducts while secondary side-branches sprout along the extending ducts [31]. The outgrowth of side branches is controlled by several hormones and signaling pathways [29]. At the end of puberty, approximately 10-12 weeks of age, TEBs reach the edge of the fat pad and disappear [31,32]. In order to determine the effect of TBX3 over-expression on the overall development of the mammary gland, we harvested the 1st and 4th mammary glands from 3 doxycycline induced double transgenic mice and from another 3 of the un-induced double transgenic littermate controls at four specific time points; 7-weeks, 10-weeks, 12-weeks of age and 10.5 days postcoitus (dpc). Mammary glands harvested at 7-weeks, 10-weeks and 12-weeks were from nulliparous mice, while those harvested at 10.5 dpc were from uniparous pregnant mice. Whole mount analysis of the 4^th ^mammary gland revealed that at 7-weeks and 10-weeks of age, TEBs in the control mice had not reached the edge of the fat pad, whereas the TEBs in doxycycline induced double transgenic mice were observed at the edge of the fat pad or had disappeared (Figure 2A, arrows indicate TEBs), suggesting that over-expression of TBX3 promotes accelerated ductal elongation. Hematoxylin and eosin staining of both the 1^st ^and 4^th ^mammary glands of the doxycycline induced double transgenic mice displayed increased primary and secondary side branching at all time points when compared to their un-induced double transgenic littermate controls (Figure 2A and 2B). We also observed an increase in tertiary side branching although this has been known to occur in response to estrous cycle [29]. In addition, pregnant doxycycline induced double transgenic mice at 10.5 dpc also displayed more alveoli tissue than the un-induced double transgenic controls (Figure 2A and 2B). The samples used for whole mount analysis were from two independent founder lines and the results were consistent between these two lines.

**Figure 3:**
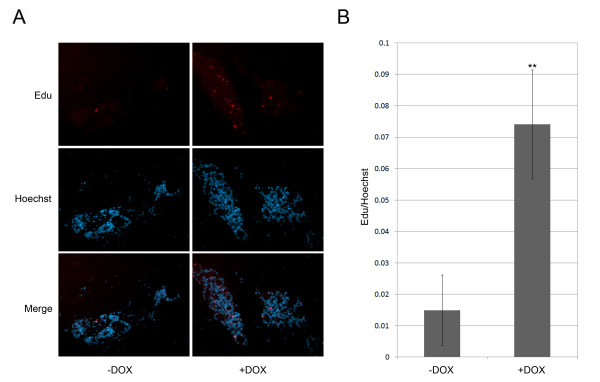
**TBX3 over-expression promotes cell proliferation**. A) Edu cell proliferation assay. The fourth mammary glands from doxycycline induced and un-induced double transgenic mice were harvested at 10.5 dpc and 5μm thick sections were embedded in paraffin and subjected to the Click-iT EdU proliferation Assay (Promega). EdU that had been incorporated into newly synthesized DNA was detected by Alexa Fluor 594 azide (red) and cell nuclei were stained with Hoechst 33342 (blue). All Images were captured at 20× magnification. B) Quantification of Edu positive cells. Fifteen random 20× fields were taken from each group of litter matched doxycycline induced and un-induced double transgenic mice. The proliferating cells were quantified and normalized to the total cell number in each field. The graph is generated from the average ratio (Edu/Hoechst) of fifteen 20× fields in each group. Error bars indicate SD; n = 1. **,P < 0.001.

Several in vitro studies have suggested that the over-expression of Tbx3/TBX3 leads to the bypass of senescence and promotes cell proliferation [8,16,21,33,34]. To determine whether the observed accelerated development of the mammary glands in TBX3 over-expressing mice is due to an increase in cell proliferation, we performed an EdU cell proliferation assay. The 4th mammary glands from pregnant doxycycline induced and un-induced double transgenic mice were harvested at 10.5 dpc and used for the assay. The proportion of nucleated cells incorporating EdU was quantified by fluorescence microscopy (Figure 3A) and normalized to the total cell number in each 20× field. After quantification, we found that the percentage of Edu positive cells is significantly (p < 000.1) higher in mammary glands over-expressing TBX3, than their un-induced controls (Figure 3B). This result suggests that over-expression of TBX3 may promote accelerated mammary gland development by promoting mammary epithelial cell proliferation *in vivo*.

Since highly proliferative tissues are associated with carcinogenesis, we next analysed the histology of the 3^rd ^mammary glands of 15 week old mice to identify if any unusual morphological changes have occurred. Hematoxylin and eosin staining of the doxycycline induced double transgenic mouse mammary gland showed mild focal hyperplasia (Figure 2B, arrows) and discontinued ductal epithelium (Figure 2B, arrowheads) when compared to the littermate control. By the age of 20 months, none of the doxycycline induced double transgenic mice had developed tumors.

### TBX3 represses *NFκBIB*

**Figure 4:**
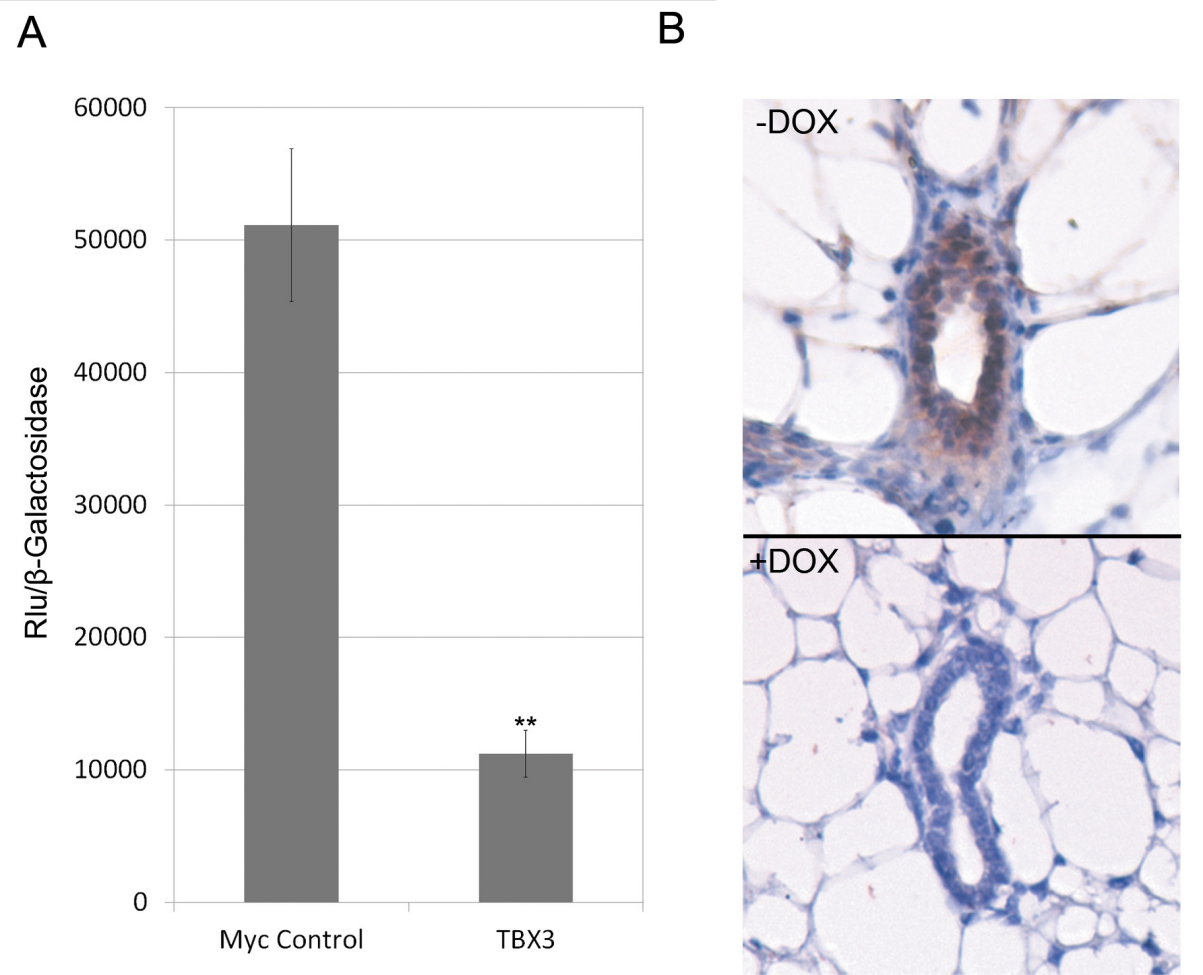
**TBX3 inhibits *NFKBIB *promoter activity**. A) Luciferase reporter assay. COS-7 cells were transfected with either pcDNA3.1-Myc (control) or pcDNA3.1-Myc-TBX3 expression vectors together with the pGL3-NFκBIB luciferase reporter construct and a β-galactosidase control plasmid (pcDNA3.1/His/LacZ). Cell lysates were harvested 48 hours after transfection and used to measure luciferase activity. β-galactosidase enzyme activity was also measured and used to normalize luciferase activity. Error bars indicate SD; n = 3. **, P < 0.001. B) Immunohistochemical staining of Nfκbib in doxycycline induced and un-induced double transgenic mice at 10 weeks of age. The third mammary glands were fixed and embedded in paraffin. Five micrometer thick sections were blocked with hydrogen peroxide and incubated with rabbit anti-Nfκbib antibody overnight. Biotinylated goat anti-rabbit IgG was used as a secondary antibody. Standard ABC kit and DAB kit were used for visualization. Nfκbib was down-regulated in the doxycycline induced double transgenic mouse as compared to its un-induced double transgenic littermate control. Images were captured at 40× magnification.

In our double transgenic mouse model in which TBX3 was over-expressed, we observed accelerated development of the mammary gland from 7 weeks of age through pregnancy; specifically enhanced branching and ductal elongation. Moreover mice that over-expressed TBX3 also had a significantly higher percentage of proliferating mammary epithelial cells than controls. Together these data suggest that TBX3 may be regulating genes that play a role in cell proliferation. Identifying the mechanism by which TBX3 promotes accelerated mammary gland development will help to further elucidate its possible role in breast cancer development. Dysregulation of the NF-κB associated pathways have been shown to play a role in breast cancer development [35]. Moreover, it has been shown that elevated NF-κB activity causes mammary hyperplasia *in vivo *[24]. Due to this observed phenotype and our previous unpublished data in which TBX3 binds to the promoter of *NFκBIB *in MCF7 cells, we investigated the role TBX3 may play in regulating the NFκB pathway. To verify that TBX3 does indeed regulate the *NFκBIB *promoter, we performed a luciferase assay. Briefly, COS-7 cells were transfected with either pcDNA3.1-Myc (control) or pcDNA3.1-Myc-TBX3 expression vector together with the pGL3-NFκBIB luciferase reporter construct and a β-galactosidase control plasmid (pcDNA3.1/His/LacZ) using Lipofectamine 2000. Forty-eight hours later, cell lysates were harvested and used to perform the luciferase assay. β-galactosidase enzyme activity was measured and used to normalize luciferase activity. The luciferase assay revealed that the activity of the *NFκBIB *promoter is significantly repressed (p < 0.0001) when TBX3 is over-expressed in COS-7 cells (Figure 4A). To determine whether the Nfκbib protein was down-regulated upon over-expression of TBX3 within the mammary gland, immunohistochemistry was performed on the mammary glands of doxycycline induced and un-induced double transgenic mice at 10 weeks of age. Staining revealed the Nfκbib expression was down-regulated in the doxycycline induced double transgenic mouse when compared to its un-induced double transgenic littermate control (Figure 4B). These data suggest that over-expression of TBX3 may promote cell proliferation within the mammary gland by repressing the expression of Nfκbib.

### Over-expression of TBX3 is associated with an increase in mammary stem-like cells

**Figure 5:**
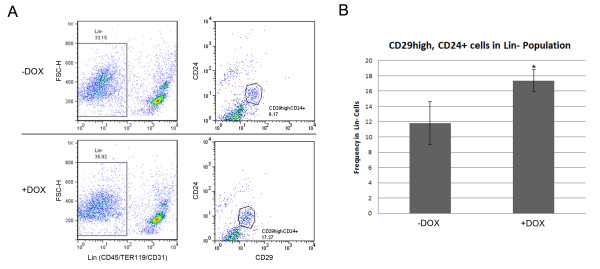
**TBX3 over-expression is associated with increased mammary stem-like cell population**. A and B) FACS analysis of mammary stem-like cells. (A) Representative graphs of gating strategies used to select Lin^- ^from Lin^+ ^cell population. Anti-CD31, anti-CD45 and anti-TER119 antibodies were used to separate the Lin^- ^and Lin^+ ^cells from harvested mammary glands of 12-week old female virgin mice (doxycycline induced and un-induced double transgenic littermates) (left panel). Gating strategy used to purify CD29^high^CD24^+ ^cells from Lin^- ^cell population (right panel). B) Statistical analysis of the average frequency of mammary stem-like cells (CD29^high^, CD24^+^) in Lin^- ^population. Two mice from each group were used to generate the data. Error bars indicate SD; n = 2, *, P < 0.05.

Another mechanism by which TBX3 over-expression may promote accelerated mammary gland development is through the proliferation of mammary stem cells. Expression of Tbx3 has been shown to promote the proliferation of breast cancer stem cells *in vitro *[36], suggesting that Tbx3 may also promote mammary stem cell proliferation. A study showed that a single Lin^-^CD24^+^CD29^high ^cell is able to generate a functional mammary gland, providing strong evidence that these cells are mammary stem cells [37]. Thus, to isolate and analyze the mammary stem-like cell population we first subtracted the mammary Lin^+ ^(CD31^+^, CD45^+ ^and TER119^+^) cells. CD31 is considered as an endothelial cell marker, and CD45 and TER119 are considered as hematopoietic cell markers [37]. Therefore, Lin^+ ^(CD31^+^, CD45^+ ^and TER119^+^) cells are considered a terminally differentiated cell population. In contrast, CD29 is a skin stem cell marker [38] and CD24 is found on neuronal stem cells [39], therefore CD29^+ ^and CD24^+ ^cells are considered mammary stem-like cells [37]. To determine whether over-expression of TBX3 promotes the proliferation of mammary stem-like cells, we dissected mammary glands from two mice at 12 weeks of age from the doxycycline induced double transgenic group and their un-induced double transgenic littermates and isolated the mammary stem-like cells using the previously mentioned cell markers. The gating strategy for Lin^- ^cells and CD24^+ ^CD29^high ^cells is shown in Figure 5A. FACs analysis revealed that over-expression of TBX3 did not affect the overall frequency of Lin^- ^cells in the mammary glands of doxycycline induced and un-induced mice, 35.92% and 33.15%, respectively (Figure 5A). However, within the Lin^- ^population, there was a significant increase in the frequency of CD24^+^CD29^high ^cells in the doxycycline induced double transgenic mice versus un-induced control; 17.37% and 9.17% respectively (p < 0.05) (Figure 5A and 5B). The average and standard deviations from both mice in each group are presented in Figure 5B. These results suggest that over-expression of TBX3 may promote proliferation of mammary stem-like cells.

## Discussion

The TBX3 T-box transcription factor plays an important role in early mammary development [3,7,12,40]. Mutations that cause haploinsufficiency of Tbx3 result in mammary gland hypoplasia in both mice and human [3,12,13]. On the other hand, Tbx3 is over-expressed in a variety of cancers, including breast cancer [16,18,26,28]. Although Tbx3 over-expression has been associated with oncogenesis by its known ability to inhibit P14^ARF ^expression and bypass senescence or by contributing to breast cancer cell migration [11,17,19], no direct evidence has been shown to suggest that over-expression of TBX3, alone, can induce tumor formation within the mammary gland. In this study, we over-expressed TBX3 within the mammary glands of mice, using a tissue-specific, doxycycline inducible transgenic system. Transgenic mouse models using constitutive promoters have provided information about specific genes and breast cancer development, particularly oncogene function [41,42]. However, there are significant limitations to these systems due to the lack of control of transgene expression. The ability to control TBX3 expression is critical since homozygous Tbx3 knockout is embryonic lethal and constitutive over-expression is potentially toxic [12,43]. We implemented a Tet-On system in our transgenic mouse model so that TBX3 transgene expression is inducible in a time and tissue-specific manner [44], enabling us to test possible TBX3 function in tumorigenesis in the mammary glands. An advantage of our mouse model is the ability to use luciferase expression as an indication of TBX3 transgene expression (Figure 1A). In this way, we are able to monitor TBX3 expression without sacrificing the animal. Using *in vivo *imaging as well as a luciferase assay, we were able to show that transgene expression is tightly controlled by doxycycline administration (Figure 1B). Our results show that this system is reliable and transgene expression could be induced in all five pairs of mammary glands.

Previous studies have shown that the five pairs of mouse mammary glands are differentially regulated by Tbx3 during early development. For example, in Tbx3 knockout studies, homozygous mutations resulted in the absence of mammary placodes, except for an occasional induction of the second pair of mammary placodes [12]. Heterozygous mutations of Tbx3 caused decreased branching morphogenesis in the first three pairs of mammary glands, but had no significant impact on the fourth and fifth pairs of mammary glands [12]. In 18.5 day old Tbx3 heterozygous embryos, 75% of the first pair of mammary glands was missing with no nipple or ductal tree formation while the second pair of mammary glands was affected to a lesser extent [21]. Although these studies suggest that Tbx3 regulates murine mammary glands differently, we found that over-expression of TBX3 promotes accelerated mammary gland development in both the first and fourth mammary glands (Figure 2A and 2B) as well as the second, third and fifth mammary glands (data not shown).

Research has solidified a role for Tbx3 in the early development of the mammary gland. Tbx3 homozygous mutant mice results in mammary gland hypoplasia while heterozygous mutations of Tbx3 caused decreased branching morphogenesis in mammary glands [12,21]. Our research complements these previous studies showing that TBX3 over-expression within the mammary glands causes hyperplasia, promoting increased secondary and tertiary branching as well as accelerated ductal elongation. It is also important to discuss that we have over-expressed human TBX3 within the mammary glands of mice. It has been shown that human TBX3 and mouse Tbx3 are 97% homologous at the protein level. Our group and others have demonstrated that human TBX3 is functional in mouse cells [9,12,16,20]. Furthermore, aTbx3 knockout mouse model was able to recapitulate the phenotype seen in humans with Ulnar Mammary Syndrome (UMS). In a study performed by Papaioannou et al., a mutation in the mouse Tbx3 gene that closely corresponds to truncation mutations seen in some individuals with UMS resulted in a deficiency in mammary placode induction and the absence or reduction of mammary buds in mutant embryos, corresponding to the mammary gland hypoplasia seen in patients with UMS. Moreover, the deficiency in the development of limb elements in individuals with UMS was also reflected in limb abnormalities in the Tbx3 mutant mice. Mutant mice had deformities in the forelimb digits, foot and fibula resulting from a failure in the development of posterior limb elements. This study exemplifies that the Tbx3 protein plays a similar role in the development of the mammary glands in both human and mice. The mechanism by which TBX3 over-expression promotes hyperplasia in mammary glands needs to be elucidated. Using an Edu cell proliferation assay, we showed that over-expression of TBX3 resulted in a dramatic increase in cell proliferation within the mammary glands of pregnant doxycycline induced double transgenic mice at 10.5 dpc (Figure 3). Although cell proliferation was not directly quantified for the other developmental time points (i.e. 7-weeks, 10-weeks, and 12-weeks), the similarity in the observed accelerated mammary gland development suggests that the increase in cell proliferation at 10.5 dpc may also play a role in causing the accelerated branching and elongation of ducts during the other phases of mammary gland growth. A study has shown that mammary epithelia lacking the gene encoding NFκBIA contained increased NFkB activity as well as increased ductal branching and widespread intraductal hyperplasia [24], similar to results seen in our study. Furthermore, aberrant activation of NF-κB increased cell proliferation and breast cancer progression [25]. In this study, we found that TBX3 inhibits the promoter activity of *NF*κ*BIB in vitro *(Figure 4A). Upon further analysis, *in vivo*, we observed that Nfκbib expression was dramatically reduced in doxycycline induced double transgenic mice as compared to its un-induced double transgenic littermate controls (Figure 4B). Taken together, our results suggest a mechanism by which TBX3 over-expression represses NFKBIB/Nfkbib expression to enhance cell proliferation and promote mammary gland hyperplasia. However, TBX3 is a multifunctional transcription factor and the NFkB pathway could be one of many pathways regulated by TBX3. Wnt signaling has also been shown to play a major role in regulating mammary gland development [27]. A TBX3^-/- ^mouse model lacked expression of LEF1 and Wnt10b [12], suggesting that Wnt signaling is a downstream target of TBX3 and that TBX3 may regulate mammary gland development via the Wnt signaling pathway. Additional experiments can be done to further elucidate other mechanisms by which TBX3 over-expression promotes mammary hyperplasia.

Studies have suggested a role for Tbx3/TBX3 in regulating the self-renewal of mouse embryonic stem (ES) cells as well as breast cancer stem-like cells [36,45-47]. Mouse ES cells require leukemia inhibitory factor (LIF) to maintain their undifferentiated state [48]. Mouse ES cells genetically modified to over-express Tbx3 and grown in culture without LIF were able to maintain their undifferentiated state [47]. Knockdown of Tbx3 expression in mouse ES cells resulted in a loss of self-renewal, causing these cells to differentiate [45]. These findings suggest that Tbx3 expression is necessary to maintain mouse ES cells in their undifferentiated state and plays a functional role to promote self-renewal. A recent study has proposed a model in which the expression of TBX3 in cancer cells promotes the expansion of cancer stem-like cells through paracrine fibroblast growth factor (FGF) signaling [36]. Over-expression of TBX3 increased the proportion of cancer stem-like cells in MCF7 cells by nine-fold as well as lead to an increase in tumorsphere formation and tumor initiation [36], suggesting that TBX3 is sufficient to promote normal and cancer stem like cell phenotypes. Due to its role in promoting proliferation of mouse ES cells and breast cancer stem-like cells as well as its requirement for early mammary gland development, TBX3 may also play a role in regulating mammary stem cell proliferation. Mammary glands consist of two cell lineages: myoepithelial and luminal epithelial cells. Both of them arise from a common progenitor, the mammary stem cell. Research has shown that a single Lin^-^CD24^+^CD29^high ^cell is able to generate a functional mammary gland, suggesting that these cells are mammary stem cells [37]. To determine whether over-expression of TBX3 affects mammary stem cell proliferation, we performed FACS analysis of the stem-like cell population, Lin^-^CD24^+^CD29^high^, in doxycycline induced double transgenic mice and their un-induced littermate controls. We found that over-expression of TBX3 significantly increased the frequency of Lin^-^CD24^+^CD29^high ^stem-like cell population (Figure 5A and 5B), indicating that TBX3 expression is associated with an increased number of mammary stem-like cells. This could explain another mechanism by which TBX3 over-expression can cause hyperplasia and accelerated mammary gland development. Further studies of the mechanisms by which TBX3 regulates mammary stem-like cells are required to improve our understanding of mammary gland development and TBX3 function.

## Conclusions

TBX3 over-expression causes mammary gland hyperplasia possibly by inhibiting *NF*κ*BIB *expression and thus promoting cell proliferation. Also, over-expression of TBX3 is associated with an increased number of mammary stem-like cells suggesting another mechanism by which TBX3 may promote mammary gland hyperplasia and contribute to breast cancer development.

## Methods

### Plasmid construction

To generate the Tet-on inducible N-myc-TBX3 expression cassette (tet-N-myc-TBX3-IRES-Luciferase), the full-length human *TBX3 *cDNA fused with the N-myc tag was subcloned from the expression vector, pcDNA-myc-TBX3, into the ClaI and SpeI sites of the TMILA plasmid, downstream of an inducible tetracycline promoter (Figure 1A). Correct insertion of the N-myc-TBX3 transgene into the TMILA plasmid was verified by sequencing.

### Generation and PCR-genotyping of transgenic mice

To generate doxycycline inducible myc-TBX3 transgenic mice, the N-myc-TBX3 expression cassette (tet-myc-TBX3-IRES-luciferase) was cut out from the TMILA-myc-TBX3 plasmid using the PvuII restriction enzyme to remove the plasmid backbone. The fragment was gel-purified using the Qiagen Gel Extraction Kit (Valencia, CA) and filtered using a 0.1 micron filter. The purified DNA fragment was then diluted with injection buffer to a 2ng/μl concentration and microinjected at the UCI Transgenic Mouse Facility. A total of 176 fertilized eggs (obtained from FVB/N egg donors that were mated with fertile males) were injected. One-hundred-sixty-five eggs were implanted in the oviducts of pseudopregnant foster mothers. From these, a total of 43 pups were obtained. Potential founders were identified by PCR-based genotyping using a pTMILA and TBX3 gene specific primer set (forward; 5'- CGCGCAATTAACCCTCACTA-3' (pTMILA), reverse; 5'-AGGAATGACCGGATCTCTCA-3' (TBX3)). A total of 8 pups carrying the N-myc-TBX3 expression cassette were used as founders to cross with established MMTV-rtTA mice to create double transgenic mice (MMTV-rtTA; tet-myc-TBX3-IRES-Luciferase).

### Doxycycline administration

Transgene expression was induced by adding 2 mg/ml doxycycline to the drinking water from weaning age (3-4 weeks) as previously described [44]. All mice involved in the experiments were examined weekly for palpable tumor formation.

### *In vivo *imaging of Tet-on inducible TBX3 luciferase reporter system

For *in vivo *mouse imaging, a cooled ICCD camera was placed on top of a light-tight box. Prior to imaging, mice were sedated by intraperitoneal injection (i.p.) of 250 ng Xylazine and 2 mg Ketamine. After 5 minutes, an aqueous solution of luciferin (BioSynth, 150 mg/ml) was injected into the peritoneal cavity at 150 mg/kg body weight. An LED light, placed around the camera, was first turned on to acquire body surface reference images. At this time the field of view (F.O.V), focus and f/stop were adjusted. Afterwards, the chamber door was closed to exclude room light. We allowed 5 minutes for the integration of the ICCD camera before images were acquired.

### Luciferase assay

To measure luciferase reporter gene expression in doxycycline induced and un-induced mammary glands of double transgenic mice, all 5 mammary glands were dissected, rinsed in PBS and tissues were homogenized in Reporter lysis buffer (Promega, Madison, WI). Insoluble tissue lysates were removed by centrifugation at 4°C for 5 minutes. Luciferase activity (Rlu) was measured using 10μl of protein lysate, the Luciferase assay kit (Promega, Madison, WI) and a Berthold luminometer (Berthold Australia Pty Ltd, Australia). The luciferase readings were normalized to total protein concentration.

### Edu proliferation assay

For assessment of cell proliferation within the mammary gland, the fourth mammary glands from doxycycline induced and un-induced double transgenic mice were harvested at 10.5 days postcoitus (dpc) and 5μm thick sections were embedded in paraffin. Cell proliferation was detected using incorporation of 5-ethynyl-2'-deoxyuridine (EdU) with the Click-iT EdU Cell Proliferation Assay Kit (Invitrogen, Camarillo, CA), following the manufacturer's instructions. EdU that had been incorporated into newly synthesized DNA was detected by Alexa Fluor 594 azide (red) and cell nuclei were stained with Hoechst 33342 (Invitrogen, Camarillo, CA). The proportion of nucleated cells incorporating EdU was determined by fluorescence microscopy (Axioskop, Zeiss, Germany). Fifteen random 20× fields were taken from each group of litter matched doxycycline induced and un-induced double transgenic mice. The proliferating cells were quantified and normalized to the total cell number in each field.

### Whole mount analysis

Whole mount preparation of mammary glands was performed at various time points as previously described [49]. Briefly, mammary glands were removed from doxycycline induced and un-induced double transgenic mice and fixed overnight in acetic acid/ethanol (1:3) solution. Fixed mammary glands were then dehydrated using 70% ethanol for 30 minutes and stained overnight with Carmine stain. The mammary glands were then destained, dehydrated through a series of washes in 70%, 95% and 100% ethanol for 30 minutes each and defatted in xylene.

### Histological staining and immunohistochemistry

The third mammary glands from doxycycline-induced and un-induced double transgenic mice were fixed and embedded in paraffin. Five micrometer thick sections were deparaffinized with xylene and stained with hematoxylin and eosin (H&E) or used for immunohistochemistry (IHC). For IHC, antigen retrieval was performed by treating deparaffinized sections with sodium citrate buffer (pH6) at 95°C for 20 minutes. The sections were then blocked for one hour with serum followed by an additional 10 minute blocking with hydrogen peroxide. Sections were incubated with rabbit anti-TBX3 (Zymed, Camarillo, CA) and rabbit anti-NFκBIB (Santa Cruz Biotechnology, Santa Cruz, CA) antibodies overnight at 4°C. The following day, sections were washed in PBS and incubated with biotinylated goat anti-rabbit IgG (Vector Laboratories, Burlingame, CA). Standard ABC kit and DAB kit (Vector Laboratories, Burlingame, CA) were used for visualization according to the manufacturer's instructions.

### *NFκBIB *promoter reporter and luciferase assay

The *NFKBIB *promoter (-2500:+500 bp) was PCR-amplified from human genomic DNA. The PCR product was digested and subcloned into the pGL3 luciferase reporter construct (Promega, Madison, WI). COS-7 cells were transfected with either pcDNA3.1-Myc (control) or pcDNA3.1-Myc-TBX3 expression vectors together with the pGL3-NF*κ*BIB luciferase reporter construct and a β-galactosidase control plasmid (pcDNA3.1/His/LacZ) using Lipofectamine 2000 (Invitrogen, Camarillo, CA). Cell lysates were harvested 48 hours after transfection. Luciferase activity was obtained using the Promega Luciferase Assay System (Promega, Madison, WI) according to the manufacturer's guidelines. β-galactosidase enzyme activity was measured using the Promega β-galactosidase Enzyme Assay System (Promega, Madison, CA) and used to normalize luciferase activity.

### Mammary epithelial cell preparation and cell sorting

Mammary epithelial cells were prepared as previously described with modifications [37]. Briefly, mammary glands were dissected and mechanically dissociated with scissors and a Tissue Tearor Homogenizer (Tearor), followed by enzymatic dissociation (DME/HAM with 5% BCS, 1 mM L-glutamine, 5μg/ml insulin, 500 ng/ml hydrocortisone, 10 ng/ml epidermal growth factor, 20 ng/ml cholera toxin, 300μg/ml collagenase, 100μg/ml hyaluranidase) for 5 hours at 37°C. Cells were pelleted by centrifugation, resuspended in 0.25% trypsin-EDTA and incubated at 37°C for 3 minutes. Cells were sequentially incubated with the following reagents: 5 mg/ml Dispase (Roche Diagnostics, Basel, Switzerland) in PBS for 5 minutes, 0.1 mg/ml DNase in PBS for 5 minutes and 0.64% NH_4_Cl for 3 minutes at 37°C. Cell suspensions were filtered through a 40-mm mesh to isolate single cells and were counted using a hematocytometer.

Mammary cells were then washed with 1 ml Buffer A (2%FBS, 0.1%NaN_3 _in PBS) and the cell pellets were resuspended in 500μl Buffer A. Twenty thousand mammary cells from each mouse were incubated with biotinylated anti-CD31, biotinylated anti-CD45 and biotinylated anti-TER119 (all 1:1000 dilution) for 15 minutes at room temperature to isolate the Lin+ cells (stained) from the Lin- cells (unstained). The cells were washed once with Buffer A and the cell pellets were resuspended in 150μl Buffer A. The cell suspension was then incubated with Streptavidin-conjugated APC, PE-labeled anti-CD24, and FITC conjugated anti-CD29 (all 1:1500) for 30 minutes at 4°C. Cells were washed twice with Buffer A and resuspended in 500μl Buffer A for analysis. (Data analysis was performed on the single cell gate using the demo version of FlowJo software http://www.flowjo.com/. Cell sorting was carried out on a Fluorescence-activated cell sorting (FACS) Vantage cell sorter (Becton Dickinson, Franklin Lakes, NJ). For all APC conjugated, PE conjugated and FITC conjugated staining, Mouse IgG (APC), Mouse IgG (PE) and Mouse IgG (FITC) isotype controls were used.

### Animal Use

Animals were maintained in an approved animal facility and all animal work was carried out in accordance with the University of California Irvine Institutional Animal Care and Use Committee (IACUC, 2002-2421)....

## Authors' contributions

TE, JL, TH conceived and designed the experiments and analyzed data. JL, TE, XS, GG, AL performed the experiments. JL, TE, TH wrote the manuscript. All authors have read and have approved the final manuscript.
